# An Evaluation of the Thermotolerance of Various Formulations of Freeze-Dried and Reconstituted Peste des Petits Ruminant Vaccines

**DOI:** 10.3390/vetsci11110525

**Published:** 2024-10-29

**Authors:** Amadou Diallo, Moipone Christina Motsoane, Hassen Belay Gelaw, Jean-De-Dieu Baziki, Cisse R. Moustapha Boukary, Gelagay Ayelet Melesse, Ethel Chitsungo, Meseret Gebresillassie, Yebechaye Degefa Tessema, Babasola O. Olugasa, Olayinka Ishola, Nick Nwankpa, Charles S. Bodjo

**Affiliations:** 1 Vaccine Production and Quality Control Program of Pan African University Life and Earth Sciences Institute (PAULESI), University of Ibadan, Ibadan 200284, Oyo State, Nigeria; peulhdiallo095@gmail.com (A.D.); moiponemotsoane3@gmail.com (M.C.M.); 2 African Union-Pan African Veterinary Vaccine Centre (AU-PANVAC), Debre Zeit P.O. Box 1746, Ethiopia; hasseng@africa-union.org (H.B.G.); bazikij@africa-union.org (J.-D.-D.B.); boukaryc@africa-union.org (C.R.M.B.); gelagaya@africa-union.org (G.A.M.); ethelc@africa-union.org (E.C.); meseretgebre@africa-union.org (M.G.); yebechayet@africa-union.org (Y.D.T.); nicknwankpa@gmail.com (N.N.); 3 Pan African University Life and Earth Sciences Institute (including Health and Agriculture), PAULESI, University of Ibadan, Ibadan 200284, Oyo State, Nigeria; bolugasa@gmail.com (B.O.O.); olayinkaishola@yahoo.com (O.I.)

**Keywords:** peste des petits ruminants vaccine, thermotolerance, stabilizers

## Abstract

Peste des Petits Ruminants (PPR) disease is widely distributed in Africa. Live attenuated PPR vaccines are produced using approved Nigeria 75/1 and Sungri/96 strains by the World Organisation of Animal Health (WOAH) to control the disease. These PPR vaccines are very efficacious; however, the main challenge is the maintaining of the cold chain during vaccine distribution and delivery. This study evaluated the thermotolerance of freeze-dried and reconstituted PPR Nigeria 75/1 vaccines from vaccine manufacturers using eight stabilizer formulations (lactalbumin hydrolysate and sucrose, sucrose and peptone, Weybridge medium, trehalose, Lactose and N-Z Amine, lactalbumin hydrolysate, sucrose and L glutamine, skimmed milk, and lactalbumin hydrolysate, maltose and gelatine). Aliquots of the reconstituted PPR vaccine batches were titrated after 2, 4, and 6 h of storage at 4 °C and 40 °C. The PPR vaccines were also titrated after storage at 40 °C and 45 °C for 3 and 5 days. The results showed that reconstituted PPR vaccine stabilized with lactalbumin hydrolysate–sucrose promoted tolerance at 40 °C for 6 h. It was also noted that all reconstituted PPR vaccine formulations except the formulation stabilized with lactalbumin hydrolysate–maltose–gelatine maintained the titre above a 10^2.5^ TCID_50_/dose after 4 h of storage at 4 °C. Furthermore, the results showed that the PPR vaccine formulation containing lactalbumin hydrolysate sucrose was as the only one that maintained the titres above 10^2.5^ TCID_50_/dose after storage at 45 °C for 5 days, with a titre loss of 10^0.95^ TCID_50_/dose. Therefore, vaccine manufacturers producing PPR vaccines for use in tropical field regions could preferably use lactalbumin hydrolysate–sucrose stabilizer in vaccine formulation.

## Simple Summary

Although there are available efficacious PPR vaccines produced using Nigeria 75/1 and Sungri/96 strains for controlling the disease, a challenge arises with the need for maintaining the cold chain during vaccine distribution and delivery since these vaccines are used in tropical areas with hot climatic conditions and are also prone to thermal degradation. This study aimed to evaluate the thermotolerance of various formulations of freeze-dried and reconstituted PPR vaccines with different formulations of stabilizers. The results obtained from this study showed that the reconstituted all PPR vaccines with saline buffer maintained the titre above 10^2.5^ TCID_50_/dose following a storage in the cold chain (at 4 °C) after 4 h, except the vaccine formulation stabilized with lactalbumin hydrolysate, maltose and gelatine. Moreover, it was noted that PPR vaccine stabilized with a formulation such as lactalbumin hydrolysate and sucrose, trehalose, and Lactose and N-Z Amine complied to the minimum titre of 10^2.5^ TCID_50_/dose after storage at 40 °C for 5 days. Therefore, the type of stabilizer used to formulate the vaccine freeze-drying process to meet the residual moisture content and the thermotolerance criteria are the keys to maintain the vaccine quality in areas with hot climatic conditions where the cold chain is difficult to maintain.

## 1. Introduction

The World Organisation of Animal Health (WOAH) has classified peste des petits ruminants (PPR) as a notifiable transboundary highly communicable viral disease of sheep and goats. It is of great economic importance, particularly in developing countries, due to its high morbidity (100%) and mortality (0–90%) [1,2]. The disease clinical signs are characterized by high pyrexia, necrosis, the erosion found in stomatitis, pneumonia, leukopenia, diarrhea, and shortness of breath [3,4]. Infection rates in enzootic areas are typically high (over 50%); during an outbreak, they can reach up to 90% [5]. PPR virus (PPRV) is a member of the *Morbillivirus* genus in the *Paramyxoviridae* family and the Mononegavirales order [6]. There are four distinct genetic lineages of PPRV strains (types I, II, III, and IV) [7].

The global veterinary community has targeted PPR as the second animal disease for eradication after the global eradication of rinderpest in 2011 [8].

An efficacious vaccine has been developed as a control tool for PPR [9,10]. PPR live attenuated vaccines manufactured using Nigeria 75/1 and Sungri/96 Strains [11,12] are the vaccines authorized by the World Organisation of Animal Health (WOAH). The World Organisation for Animal Health recommended that the required minimum titre per dose of the PPR Nigeria 75/1 vaccine is 10^2.5^ TCID_50_. The main challenge of the use of these vaccines is the need to maintain the cold chain [13]. It is live attenuated vaccines which are generally in tropical and subtropical areas affected by high temperatures and poor infrastructures [14].

Heat-stable vaccines are a promising way to simplify vaccine distribution for an efficient immunization campaign [15]. The thermostability of the PPR vaccine is crucial, and high ambient temperatures can affect its effectiveness. Stabilizers and their formulation for freeze-drying can improve vaccine stability [16]. PPR vaccines produced by different manufacturers are freeze-dried forms using various stabilizer formulations. Several studies have revealed that PPR vaccines stabilized with trehalose and sucrose stabilizers maintained titres above 10^2.5^ TCID_50_/dose after exposure at 40 °C for 5 days [17].

There is a need to investigate the stability of such vaccines before and after reconstitution. The objective of this study was to evaluate the thermostability of various formulations of freeze-dried and reconstituted PPR vaccines after storage at 4 °C, 40 °C, and 45 °C.

## 2. Materials and Methods

### 2.1. Study Area

This research was conducted at the African Union–Pan African Veterinary Vaccine Centre in Bishoftu (AU-PANVAC), which is the only institution in Africa mandated to provide independent quality control of veterinary vaccines.

### 2.2. Selection of PPR Vaccine Batches

PPR vaccine batches submitted by manufacturers to the AU-PANVAC for routine quality control were selected based on the stabilizer formulations used for freeze-drying (Table 1). These vaccines were kept at the recommended storage condition (−20 °C) until testing. The residual moisture content of the vaccine batches was also taken into consideration.

### 2.3. Preparation of Diluent

To produce normal saline, 8.5 g of sodium chloride was diluted in 1000 millilitres of distilled water (0.85%). The diluent was dispensed in a 100 mL volume and autoclaved at 121 °C for 30 min. Aliquots were kept at the recommended storage conditions (2–8 °C) until use [17].

### 2.4. Vaccine Storage, Reconstitution, and Incubation

Selected PPR vaccine vials (listed in Table 1) stored at the recommended conditions (−20 °C) were incubated at 40 °C and 45 °C for 3 and 5 days for titration. Vaccine vials stored at −20 °C and incubated at 40 °C and 45 °C were reconstituted with 100 mL of normal saline for titration. In addition, aliquots from reconstituted vaccine vials stored at −20 °C were also incubated at 4 °C and 40 °C for 2, 4, and 6 h for titration. The data loggers were placed in each incubator for temperature recording.

### 2.5. Preparation of Cells

African green monkey kidney (Vero) cells (ECACC origin) were cultivated in Glasgow’s minimum essential medium (GMEM) supplemented with 10% foetal calf serum (GIBCO) and 1% mixed antibiotic antimycotic solution (GIBCO). A precultured 175 cm^2^ flask of Vero cells at 80–100% confluence was used for passage. A cell suspension at a concentration of 1.5 × 10^5^ cells/mL was prepared for titration.

### 2.6. PPR Vaccine Titration

Vaccine titrations were performed in Vero cells in 96-well microtitre culture plates [18,19]. The freeze-dried vaccine vial was reconstituted with 2 mL of phosphate-buffered saline, while the reconstituted vaccine was directly diluted serially. Tenfold serial dilutions (10^−1^ to 10^−8^) of samples in GMEM with no foetal calf serum were made ready. One hundred microliters of each dilution was dispensed into the microplate to have 10 replicates per dilution (10^−1^ in wells A1–A10, 10^−2^ in wells B1–B10, and 10^−8^ in wells H1–H10). One hundred microlitres of GMEM with no foetal calf serum was dispensed in columns 11 and 12 to serve as uninoculated controls. Each batch for each storage condition and each day was titrated in duplicate. A PPR vaccine control with a known titre was included in each run. All the test plates were incubated at 37 °C in a humidified incubator with 5% CO_2_ for 10 to 12 days. The plates were checked for cytopathic effect (CPE) under an inverted microscope, starting from day 3 to the final day 12, and the titres were calculated according to the Spearman Karber formula [20].

### 2.7. Data Analysis

Data entry and management from the potency study were conducted using Microsoft Office Excel (Version 2408 Build 16.0.17928.20114). The effects were reported to be statistically significant at *p* values less than 5% (*p* < 0.05). The laboratory data were entered into a Microsoft Excel spreadsheet, analysed and compared for thermotolerance.

## 3. Results

The evaluation of thermotolerance of the freeze-dried PPR vaccine formulations with various types of stabilizers was conducted after incubation at 40 °C and 45 °C for 3 and 5 days. To evaluate the stability of the reconstituted vaccine, the aliquot of reconstituted vials with saline buffer were incubated at 4 °C and 40 °C for 2, 4, and 6 h for titration. The titration of the aliquot immediately after vaccine reconstitution was conducted as titre at 0 h.

### 3.1. Residual Moisture Results

Freeze-drying has been used successfully to preserve and store vaccines, microbial cultures, and other labile biological products. The residual moisture content in the lyophilized PPR vaccine batches selected were below 3.5% (Table 2), which complies to the requirement according to the WOAH standard.

### 3.2. The Stability of the Vaccine Batches

#### 3.2.1. Stability of Freeze-Dried PPR Vaccine Formulations at 40 °C and 45 °C

**Figure 1:**
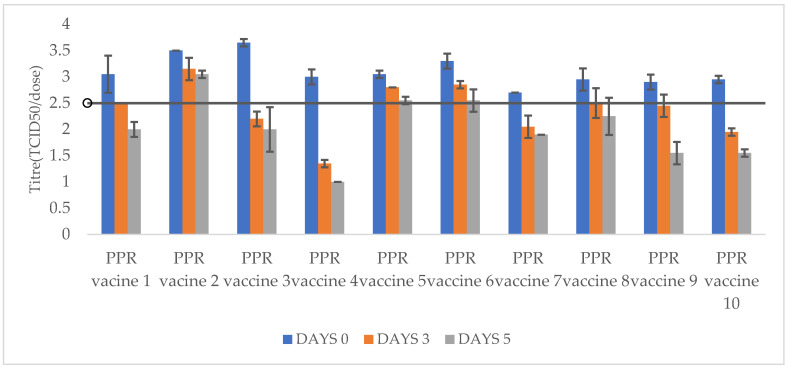
Titres of PPR freeze-dried vaccines after storage at 40 °C for 3 and 5 days. All PPR vaccines had titres above 10^2.5^ TCID_50_/dose at day 0. PPR vaccines (2, 5, 6) had minimum required titre of 10^2.5^ TCID_50_/dose after storage at 40 °C for 3 and 5 days while PPR vaccines (1, 3, 4, 7, 8, 9, and 10) had titres below 10^2.5^ TCID_50_/dose after storage at 40 °C for 3 and 5 days.

**Figure 2:**
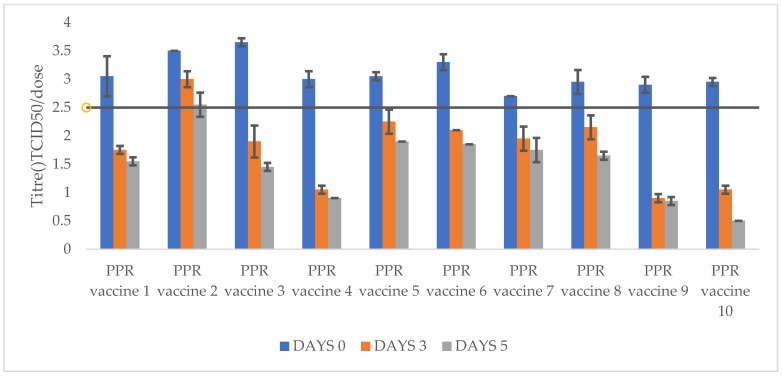
Titres of PPR freeze-dried vaccines after at storage at 45 °C for 3 and 5 days. All PPR vaccines had titres above 10^2.5^ TCID_50_/dose at day 0. Only PPR vaccine 2 maintained minimum required titre of 10^2.5^ TCID_50_/dose after storage at 45 °C for days 3 and 5. However, PPR vaccines 1, 3, 4, 5, 6, 7, 8, 9, and 10) had titres below 10^2.5^ TCID_50_/dose after storage at 45 °C for 3 and 5 days.

The titres of freeze-dried PPR vaccine formulations after incubation at 40 °C and 45 °C for 3 and 5 days is presented in Table 2 as well as Figure 1 and Figure 2.

Three PPR freeze-dried vaccine formulations stabilized with lactalbumin hydrolysate sucrose (PPR vaccine 2), trehalose (PPR vaccine 5), and Lactose and N-Z Amine (PPR vaccine 6) showed good thermotolerance, maintaining titres above 10^2.5^ TCID_50_/dose (with titre losses a maximum of 10^0.75^ TCID_50_/dose) after 5 days incubation at 40 °C.

PPR freeze-dried vaccine stabilized with lactalbumin hydrolysate–sucrose (PPR vaccine 1) maintained a titre of 10^2.5^ TCID_50_/dose (with a titre drop of 10^0.55^) only up to 3 days incubation at 40 °C. The remaining freeze-dried vaccine formulations (PPR vaccines 3, 4, 7, 8, 9, and 10) showed titres below 10^2.5^ TCID_50_/dose throughout the incubation time of the experiment at 40 °C.

For the incubation at 45 °C, only the PPR vaccine 2 (stabilised with lactalbumin hydrolysate and sucrose) maintained a titre above 10^2.55^ TCID_50_/dose (with a titre drop of 10^0.95^ TCID_50_/dose) for 5 days. The remaining freeze-dried vaccine formulations (PPR vaccines 1, 3, 4, 5, 6, 7, 8, 9, and 10) showed titres below 10^2.5^ TCID_50_/dose from after 3 days incubation.

#### 3.2.2. Stability of Reconstituted PPR Vaccine Formulations at 4 °C and 40 °C

**Figure 3:**
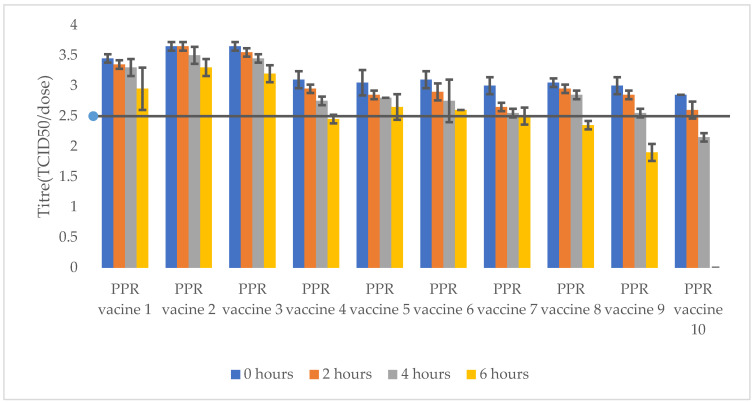
Titres of reconstituted PPR vaccines after storage at 4 °C for 2, 4, and 6 h. PPR vaccines 1 to 10 had titres above 10^2.5^ TCID_50_/dose at 0 h. PPR vaccines (1, 2, 3, 5, and 6) maintained titres above 10^2.5^ TCID_50_/dose up to 6 h. PPR vaccines (4, 7, 8, and 9) maintained titre of 10^2.5^ TCID_50_/dose up to 4 h. PPR vaccine 10 maintained titre of 10^2.5^ TCID_50_/dose only up to 2 h of storage.

**Figure 4:**
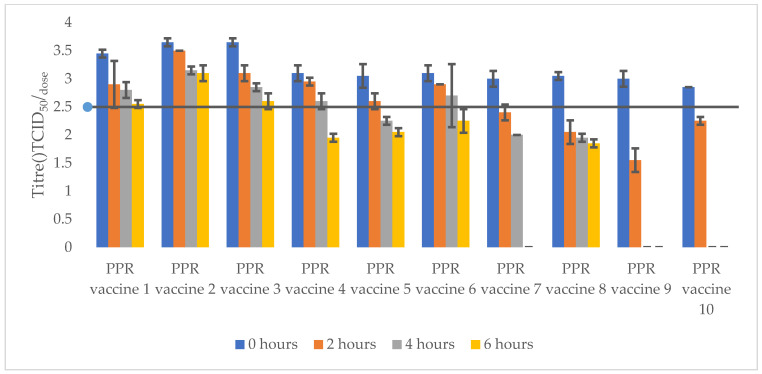
Titres of PPR reconstituted vaccines after storage at 40 °C for 2, 4, and 6 h. All PPR vaccines had titres above 10^2.5^ TCID_50_/dose at 0 h. PPR vaccines 2 and 3 maintained titre above 10^2.5^ TCID_50_/dose up to 6 h of incubation. PPR vaccines 1, 4, and 6 had titres above 10^2.5^ TCID_50_/dose up to 4 h of incubation. PPR vaccines 7, 8, 9, and 10 could not maintain minimum titre of 10^2.5^ TCID_50_/dose after storage at 40 °C for 2, 4, and 6 h.

Ten PPR vaccine batches from the AU-PANVAC repository were tested. The stability of PPR reconstituted vaccine batches in saline water was evaluated after incubation at 4 °C and 40 °C for 2, 4, and 6 h. The acceptance criterion for the vaccine titre was ≥10^2.5^ TCID_50_/dose, as per the WOAH manual on diagnostic tests and vaccines. The data are presented in Table 3, and Figure 3 and Figure 4.

- *The PPR vaccines reconstituted with a diluent and incubated at 4 °C for 2, 4, and 6 h are classified into three groups based on the titration results, as follows:*
  - Reconstituted PPR vaccines maintaining a titre above 10^2.5^ TCID_50_/dose and showing a titre drop of less than 10^0.5^ TCID_50_ after storage at 4 °C for 6 h: these batches were freeze dried using stabilizers including lactalbumin hydrolysate–sucrose (PPR vaccines 1 and 2), sucrose–peptone (PPR vaccine 3), trehalose (PPR vaccine 5), Lactose and N-Z Amine (PPR vaccine 6), and lactalbumin hydrolysate–sucrose–L glutamine (PPR vaccine 7).
  - Reconstituted PPR vaccines maintaining a titre above 10^2.5^ TCID_50_/dose up to 4 h as maximum after incubation at 4 °C: this group included vaccine batches freeze dried using stabilizers including Weybridge medium (PPR vaccine 4), skimmed milk (PPR vaccine 8), and Lactose and N-Z Amine (PPR vaccine 9).
  - One reconstituted PPR vaccine freeze dried using lactalbumin hydrolysate, maltose and gelatine (PPR vaccine 10) stabilizer maintained the titre above 10^2.5^ TCID_50_/dose up to 2 h of incubation at 4 °C but failed at 4 h.
- *PPR vaccines reconstituted with a diluent and incubated at 40 °C for 2, 4, and 6 h are classified into three groups based on the titration results, as follows:*
  - Reconstituted PPR vaccines maintaining a titre above 10^2.5^ TCID_50_/dose for up to 6 h of incubation at 40 °C: these vaccines showed excellent thermotolerance, with a titre loss below 10^1.0^ TCID_50_/dose and used stabilisers such as lactalbumin hydrolysate–sucrose (PPR vaccine 1, PPR vaccine 2) and sucrose–peptone (PPR vaccine 3).
  - Reconstituted PPR vaccines maintaining a titre above 10^2.5^ TCID_50_/dose up to 4 h as maximum: these vaccines showed relative thermotolerance and use stabilizers such as Weybridge medium (PPR vaccine 4) and Lactose and N-Z Amine (PPR vaccine 6).
  - The reconstituted PPR vaccine 5 using the trehalose stabilizer managed to maintain its titre above 10^2.5^ TCID_50_/dose up to 2 h but failed for 4 and 6 h of incubation.
  - However, reconstituted PPR vaccine batches stabilized with lactalbumin hydrolysate–sucrose–L glutamine (PPR vaccine 7), skimmed milk (PPR vaccine 8), Lactose Monohydrate and N-Z Amine (batches PPR vaccine 9), and lactalbumin hydrolysate–maltose–gelatine (PPR vaccine 10) failed to maintain a titre above 10^2.5^ TCID_50_/dose, even at 2 h of incubation.

### 3.3. Accuracy of Results

**Figure 5:**
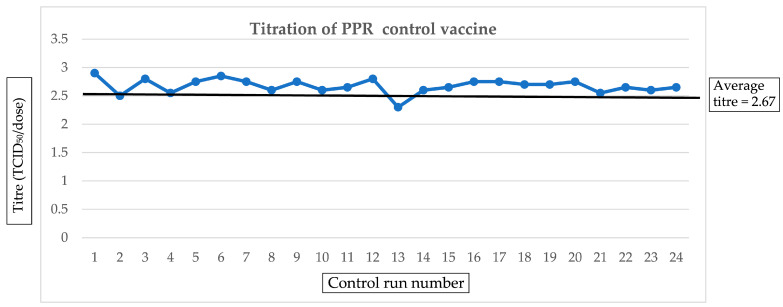
The performance of PPR vaccine control (TCID_50_). The titres from control runs 1, 2, 3, 4, 5, 6, 7, 8, 9, 10, 11, 12, and 14 to 24 had the minimum required titre of 10^2.5^ TCID_50_/dose. An exception was seen with control run 13, with the titre slightly below 10^2.5^ TCID_50_/dose.

A total of 40 plates were used for the control vaccine in this study. The accuracy of the vaccine titres was determined using a coefficient of variability (CV) with a formula of $CV=\frac{s}{\bar{x}}*100\%$. The coefficient of variability was 4.1% (Figure 5), which was considered to indicate the good accuracy (<5%) of the tests conducted on the 48 controls.

## 4. Discussion

Live attenuated vaccines are more susceptible to potency loss during storage and delivery, posing a substantial challenge in managing viral infections. These vaccines require improved excipient formulations to offer adequate stability during long-term storage. The present study investigated the thermotolerance of reconstituted and freeze-dried PPR vaccine formulations stored under different temperature conditions. In this study, PPR vaccine formulations stabilized with lactalbumin hydrolysate–sucrose, sucrose–peptone, Weybridge medium, trehalose, Lactose and N-Z Amine, lactalbumin hydrolysate–sucrose–L glutamine, skimmed milk, and lactalbumin hydrolysate, maltose and gelatine were tested.

Most of the diluents that have been used for the reconstitution of PPR vaccines are normal saline, trehalose diluent, 1 M MgSO_4_, the conventional PBS, and the diluent MgCl. Previous studies have revealed that normal saline is the best of all the diluents used for the reconstitution of PPR vaccines [21]. In the present study, normal saline was used as a diluent to reconstitute the PPR vaccine formulations. Each of the reconstituted vaccine batches were aliquoted and kept at 4 °C and 40 °C. Titrations of aliquots from each storage condition were done at 0 h as well as 2, 4, and 6 h post-incubation. All the vaccine formulations maintained a titre above the 10^2.5^ TCID_50_/dose up to 4 h of storage at 4 °C after reconstitution, complying to WOAH standards, except the vaccine stabilized with lactalbumin hydrolysate, maltose and gelatine which maintained the titre above 10^2.5^ TCID_50_/dose only up to 2 h of storage at 4 °C. Furthermore, PPR vaccine formulations stabilized with lactalbumin hydrolysate and sucrose; sucrose and peptone; trehalose; Lactose and N-Z Amine; and lactalbumin hydrolysate, sucrose and L glutamine maintained a titre above 10^2.5^ TCID_50_/dose after 6 h of storage at 4 °C. This study therefore revealed that the PPR vaccine reconstituted in a saline buffer could be used in the field for up to 4 h without significant degradation and the cold chain should be maintained. On the other hand, vaccine formulations stabilized with lactalbumin hydrolysate and sucrose, sucrose and peptone, Weybridge medium maintained the minimum titre of 10^2.5^ TCID_50_/dose up to 4 h of storage at 40 °C. It was a bit surprise to obtain one PPR vaccines stabilized with lactalbumin hydrolysate and sucrose, and the sucrose and peptone maintaining a titre above 10^2.5^ TCID_50_/dose after incubation at 40 °C for 6 h.

Freeze-dried vaccine vials were incubated for 3 days and 5 days at 40 °C and 45 °C and titrations were undertaken thereafter. Based on the agreed minimum requirements at the FAO workshop in Rome in December, 2017, a thermotolerant PPR vaccine must be able to maintain the minimum required titre of 10^2.5^ TCID_50_/dose after storage at 40 °C for 5 days. Data presented showed three PPR vaccines batches (PPR vaccines 2, 5, and 6) with a titre above 10^2.5^ TCID_50_/dose after storage at 40 °C for 5 days. These vaccines use stabilizers such as lactalbumin hydrolysate–sucrose, trehalose, and Lactose and N-Z Amine and presented an initial titre greater than 1 log10 compared with the required value of 10^2.5^ TCID_50_/dose. These stabilizers act as cryoprotectants that protect the virus from heat stress shock [22]. The thermo-degradation of the PPR vaccines 2, 5, and 6 indicates a maximum titre drop of 0.75 log10. In addition, the PPR vaccine 2 stabilized with lactalbumin hydrolysate–sucrose maintained a titre above 10^2.5^ TCID_50_/dose, with a drop in titre of 10^0.95^ TCID_50_/dose after incubation at 45°C for 5 days. The PPR vaccine 1, which was also stabilized with lactalbumin hydrolysate–sucrose, maintained a titre of 10^2.5^ TCID_50_/dose only up to day 3 at 40 °C and failed to maintain the required titre on day 5 at 40 °C as well as on days 3 and 5 at 45 °C. This could be due to the higher moisture content of 2.5% in PPR vaccine 1 compared to 1.1% for PPR vaccine 2. All the remaining PPR vaccine formulations stabilized with sucrose–peptone, Weybridge medium, lactalbumin hydrolysate–sucrose–L glutamine, skimmed milk, and lactalbumin hydrolysate–maltose–gelatine did not maintain a titre above 10^2.5^ TCID_50_/dose at 40 °C after 5 days of incubation. Similar results were reported in previous studies conducted [22,23]. Discrepancies were obtained between the titre drops of the two batches of lactalbumin hydrolysate–sucrose (PPR vaccine 1 and PPR vaccine 2) and Lactose and N-Z Amine (PPR vaccine 6 and PPR vaccine 9). This could be due to the difference in the freeze-drying process between manufacturers, which can also have an impact due to the residual moisture contents.

One of the crucial elements in the quality control of lyophilized vaccines is analysing the residual moisture content. A higher percentage of residual moisture causes the vaccine to be unstable, which in turn results in a decreased shelf life [24]. Freeze-dried vaccine vials must have a residual moisture content that is less than or equal to 3.5% according to the WOAH [18]. In the present study, the residual moisture levels for all freeze-dried vaccine batches ranged from 1.1 to 3.5%.

## 5. Conclusions

Ensuring the stability of vaccines is important for successful global immunization programs. The results of this study suggested that freeze-dried PPR vaccine stabilized with formulations such as lactalbumin hydrolysate–sucrose, trehalose, and Lactose and N-Z Amine complied to the minimum titre of 10^2.5^ TCID_50_/dose after storage at 40 °C for 5 days, currently used at the AU-PANVAC as the criteria for the evaluation of PPR thermotolerant vaccines. Further investigation needs to be conducted to refine these criteria, taking into account the drop in titre, as some vaccines might initially have a high titre. Furthermore, it was noted that the vaccine constituted with saline buffer, except the formulation stabilized with lactalbumin hydrolysate–maltose–gelatine, maintained a titre above 10^2.5^ TCID_50_/dose following storage in a cold chain (at 4 °C) after 4 h. Therefore, the type of stabilizer used to formulate the vaccine and freeze-drying process to meet the residual moisture content and the thermotolerance criteria are the keys to maintaining vaccine quality in areas with hot climatic conditions where the cold chain is difficult to maintain.

## Figures and Tables

**Table 1:** List of PPR vaccine batches and stabilizers used in the study.

Batch No.	Stabilizer Used
PPR Vaccine 1	Lactalbumin hydrolysate–sucrose
PPR Vaccine 2	Lactalbumin hydrolysate–sucrose
PPR Vaccine 3	Sucrose–peptone
PPR Vaccine 4	Weybridge medium
PPR Vaccine 5	Trehalose
PPR Vaccine 6	Lactose and N-Z Amine
PPR Vaccine 7	Lactalbumin hydrolysate-sucrose–L glutamine
PPR Vaccine 8	Skimmed milk
PPR Vaccine 9	Lactose and N-Z Amine
PPR Vaccine 10	Lactalbumin hydrolysate–haltose–gelatine

**Table 2:** Titres of freeze-dried PPR vaccine batches after incubation at 40 °C and 45 °C for 3 and 5 days. Residual moisture content.

Vaccines Batches Tested	Vaccine Titre (TCID_50_/dose)										Residual Moisture Content	
	−20 °C		40 °C				45 °C					
	Day 0		Day 3		Day 5		Day 3		Day 5			
	Titre	SD (+/−)	Titre	SD (+/−)	Titre	SD (+/−)	Titre	SD (+/−)	Titre	SD (+/−)	(%)	SD (+/−)
PPR Vaccine 1	3.05	0.35	2.5	0.00	2	0.14	1.75	0.07	1.55	0.07	2.5	0.22
PPR Vaccine 2	3.5	0.00	3.15	0.21	3.05	0.07	3	0.14	2.55	0.21	1.1	0.35
PPR Vaccine 3	3.65	0.07	2.2	0.14	2	0.42	1.5	0.28	1.45	0.07	1.9	0.34
PPR Vaccine 4	3	0.14	1.35	0.07	1	0.00	1.05	0.07	0.9	0.00	3.5	0.00
PPR Vaccine 5	3.05	0.07	2.8	0.00	2.55	0.07	2.25	0.21	1.9	0.00	2.2	0.26
PPR Vaccine 6	3.3	0.14	2.85	0.07	2.55	0.21	2.1	0.00	1.85	0.00	1.7	1.01
PPR Vaccine 7	2.7	0.00	2.05	0.21	1.9	0.00	1.95	0.21	1.75	0.21	2.4	0.00
PPR Vaccine 8	2.95	0.21	2.5	0.28	2.25	0.35	2.15	0.21	1.65	0.07	2.2	0.38
PPR Vaccine 9	2.9	0.14	2.45	0.21	1.55	0.21	0.9	0.14	0.85	0.07	2.7	0.13
PPR Vaccine 10	2.95	0.07	1.95	0.07	1.55	0.07	1.05	0.07	0.5	0.00	1.9	0.11

**Table 3:** Titres (log10) TCID_50_ of batches of PPR reconstituted vaccine after incubation at 4 °C and 40 °C for 2, 4, and 6 h.

Batch No.	−20 °C		Reconstituted Vaccine Titre (TCID_50_/dose) Following Incubation at 4 °C						Reconstituted Vaccine Titre (TCID_50_/dose) Following Incubation at 40 °C					
	0 h		2 h		4 h		6 h		2 h		4 h		6 h	
	Titre	SD (+/−)	Titre	SD (+/−)	Titre	SD (+/−)	Titre	SD (+/−)	Titre	SD (+/−)	Titre	SD (+/−)	Titre	SD (+/−)
PPR Vaccine 1	3.45	0.07	3.35	0.07	3.3	0.14	2.95	0.35	2.9	0.42	2.8	0.14	2.55	0.07
PPR Vaccine 2	3.65	0.07	3.65	0.07	3.5	0.14	3.3	0.14	3.5	0.00	3.15	0.07	3.1	0.14
PPR Vaccine 3	3.65	0.07	3.55	0.07	3.45	0.07	3.2	0.14	3.1	0.14	2.85	0.07	2.6	0.14
PPR Vaccine 4	3.1	0.14	2.95	0.07	2.75	0.07	2.45	0.07	2.95	0.07	2.6	0.14	1.95	0.07
PPR Vaccine 5	3.05	0.21	2.85	0.07	2.8	0.00	2.65	0.21	2.6	0.14	2.25	0.07	2.05	0.07
PPR Vaccine 6	3.1	0.14	2.9	0.14	2.75	0.35	2.6	0.00	2.9	0.00	2.7	0.56	2.25	0.21
PPR Vaccine 7	3	0.14	2.65	0.07	2.55	0.07	2.5	0.14	2.4	0.14	2.0	0.00	-	-
PPR Vaccine 8	3.05	0.07	2.95	0.07	2.85	0.07	2.35	0.07	2.05	0.21	1.95	0.07	1.85	0.07
PPR Vaccine 9	3	0.14	2.85	0.07	2.55	0.07	1.9	0.14	1.55	0.21	-	-	-	-
PPR Vaccine 10	2.85	0.00	2.6	0.14	2.15	0.14	-	0.00	2.25	0.07	-	-	-	-

## Data Availability

The data utilized in this study can be made available upon valid request.
